# The NLRP3 inflammasome – interleukin 1β axis in uveal melanoma

**DOI:** 10.1002/2211-5463.13566

**Published:** 2023-02-12

**Authors:** Victor S. M. C. Correa, Nikolaos E. Efstathiou, Dimitrios P. Ntentakis, Zhen Yu, Toshio Narimatsu, Evangelos Gragoudas, Ivana K. Kim, Demetrios G. Vavvas

**Affiliations:** ^1^ Retina Service, Ines and Fred Yeatts Retina Research Laboratory, Angiogenesis Laboratory, Department of Ophthalmology Massachusetts Eye and Ear, Harvard Medical School Boston MA USA

**Keywords:** IL‐1, inflammasome, melanoma, NLRP3, skin, uveal

## Abstract

Uveal melanoma (UM) is the most common primary intraocular cancer in the adult population. Recent studies suggested that the NLRP3 inflammasome could be a therapeutic target for cutaneous melanoma (CM), but the role of NLRP3 in UM remains unknown. Here, we analyzed the NLRP3‐IL‐1β axis in 5 UM and 4 CM cell lines. Expression of NLRP3 mRNA in UM and CM was low, and expression in UM was lower than in CM (*P* < 0.001). NLRP3 protein levels were below detection limit for all cell lines. UM exhibited lower baseline IL‐1β secretion than CM, especially when compared to the Hs294t cell line (*P* < 0.05). Bioinformatic analysis of human tumor samples showed that UM has significantly lower expression of NLRP3 and IL‐1β compared with CM. In conclusion, our work shows evidence of extremely low NLRP3 expression and IL‐1β secretion by melanoma cells and highlight differences between CM and UM.

## Graphical abstract

The NLRP3 inflammasome–Interleukin‐1β axis was recently proposed as a therapeutic target for cutaneous melanoma. Here, we show that NLRP3 and IL‐1β levels in uveal melanoma are minimal. These findings might contribute to future melanoma translational research.

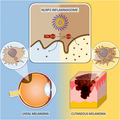

**Abbreviations**
CM: Cutaneous melanoma
ELISA: Enzyme‐linked immunosorbent assay
IL‐1β: Interleukin‐1β
NLRP3: Nucleotide‐binding, leucine‐rich repeat and pyrin domain‐containing protein 3
qPCR: Quantitative polymerase chain reaction
RPE: Retinal pigment epithelium
TCGA: The Cancer Genome Atlas
UM: Uveal melanoma
WB: Western blotting

Uveal melanoma (UM) is the most common primary intraocular cancer in the adult population, with an incidence of around 7 per million per year [1, 2]. It can arise from melanocytes of any part of the uveal tract, but affects the posterior choroid in more than 90% of cases [3]. Uveal melanoma is slowly growing tumor, and less than 2% of patients have detectable systemic metastasis at presentation [4]. Despite effective primary treatment, more than half of the patients will develop metastatic disease [5, 6], and once a metastasis is diagnosed, the median survival drops to 6–12 months [5, 7, 8, 9]. Great progress has been made in the past decade for advanced cutaneous melanoma (CM) with immunotherapy, such as immune checkpoint inhibitors [10, 11], reducing the overall mortality rate by 18% and doubling the 5‐year survival rate for metastatic CM from 15% to 30% between 2011 and 2017 [12, 13]. On the contrary, such therapies have not been able to help patients with advanced UM [9, 14, 15, 16, 17, 18, 19, 20, 21], for which the median survival after metastatic spread has remained largely unchanged over the past 40 years [7, 22]. This highlights the intrinsic differences between these tumors and the need to better understand UM immune microenvironment and explore new treatment strategies for UM.

Recently, nucleotide‐binding, leucine‐rich repeat and pyrin domain‐containing protein 3 (NLRP3) inflammasome has been suggested as a new therapeutic target for CM [23, 24]. NLRP3 inflammasome is a multimeric intracellular protein complex involved in the innate immune response and is assembled and activated following damage‐associated molecular patterns (DAMPs) or pathogen‐associated molecular patterns (PAMPs) signaling, which promotes caspase‐1 activation and the release of the pro‐inflammatory interleukin‐1β (IL‐1β) and interleukin‐18 (IL‐18), and ultimately leads to cell death through pyroptosis [25]. Tengesdal *et al*. [23, 24] showed that CM cell lines had active NLRP3‐IL1β inflammasome axis aiding tumor‐induced immunosuppression, and inhibition of NLRP3 could suppress melanoma growth *in vivo* in animal models. However, it is not known if this axis is present in UM as well. Here, we analyzed the baseline NLRP3‐IL‐1β axis in 5 different UM cell lines and contrasted them to four CM cell lines. We found that UM cells had extremely low levels of NLRP3 and IL1β. Furthermore this was confirmed in bioinformatic analysis of human samples, suggesting that in contrast to the work in CM, this may not be a promising target for future therapies of UM.

## Materials and methods

### Cell culture

Human primary uveal melanoma cell lines (92.1, Mel270, and Mel285), human uveal melanoma liver metastasis cell lines (OMM2.3 and MM28), primary cutaneous melanoma cell lines (OCM3 and A375), and cutaneous melanoma lymph node metastasis cell lines (Hs294t and Sk‐Mel28) were studied. Cell lines mutational profile and original references are summarized on Table S1. OMM2.3, Mel 270, and Mel 285 cell lines were kindly provided by Dr Bruce R. Ksander (Schepens Eye Research Institute, Boston, MA, USA). MM28 and OCM3 cell lines were kindly provided by Dr Martine J. Jager (Leiden University Medical Center, Leiden, the Netherlands). SK‐Mel28, Hs294t, and A375 cell lines were purchased from ATCC (#HTB‐72, #HTB‐140, #CRL‐1619, ATCC, Manassas, VA, USA). Cell line 92.1 was purchased from Millipore Sigma (#13012458‐1VL, Millipore Sigma, Burlington, MA, USA). Cells were cultured in a 37 °C humidified incubator under 5% CO_2_ atmosphere. Mel285 and Mel270 were cultured in RPMI 1640 medium (#11875093, Thermo Fisher Scientific, Waltham, MA, USA) supplemented with 1% nonessential amino acids (#11140050, Thermo Fisher Scientific), 1% vitamin solution (#11120052, Thermo Fisher Scientific), 100 U·mL^−1^ penicillin, 100 U·mL^−1^ streptomycin (#15140122, Thermo Fisher Scientific), and 10% heat‐inactivated FBS (#10438026, Thermo Fisher Scientific). 92.1, OCM3, OMM2.3, and THP‐1 were cultured in RPMI 1640 medium supplemented with 100 U·mL^−1^ penicillin, 100 U·mL^−1^ streptomycin, and 10% heat‐inactivated FBS. MM28 was cultured in IMDM (#12440053, Thermo Fisher) supplemented with 200 U·mL^−1^ penicillin, 200 U·mL^−1^ streptomycin, and 20% heat‐inactivated FBS. Hs294t and A375 were cultured in DMEM medium (#11965092, Thermo Fisher Scientific) supplemented with 100 U·mL^−1^ penicillin, 100 U·mL^−1^ streptomycin, and 10% FBS. Sk‐Mel28 was cultured in EMEM medium (#30‐2003, ATCC) supplemented with 100 U·mL^−1^ penicillin, 100 U·mL^−1^ streptomycin, and 10% FBS. Cells were passaged by trypsinization with 0.25% EDTA trypsin (#25200056, Thermo Fisher Scientific) when they reached 90% confluency using sterile technique. Medium was changed every 2–3 days.

### ELISA

Cells were seeded in 12‐well plate at 10^5^ cells per well and cultured for 48 h until confluency (#665180, Greiner Bio‐One, Monroe, NC, USA). Media were then aspirated and 500 μL of fresh media was added, and cells were incubated for another 24 h. Supernatant was used for human IL‐1β quantification using Quantikine ELISA kit (#DLB50, R&D Systems, Minneapolis, MN, USA), according to the manufacturer's protocol. Plate was read with SpectraMax 190 absorbance microplate reader (Molecular Devices, San Jose, CA, USA) using the softmax pro software v5.4.1 (Molecular Devices). IL‐1β quantification was measured five times per cell line.

### Western blotting (WB)

Cells were seeded in 6‐well plate (#657160, Greiner Bio‐One) at 2.5 × 10^5^ cells per well and cultured for 48 h until confluency with 2 mL of media per well. After reaching confluency, wells were washed with serum‐free media and then stored overnight at −80 °C. THP‐1‐derived macrophage was obtained by incubating THP‐1 cells (5 × 10^5^ cell per well in 6‐well plate) overnight with 80 nm of phorbol 12‐myristate 13‐acetate (#P1585, Millipore Sigma) and subsequently washed with serum‐free media and stored overnight at −80 °C. Next day, 150 μL of prechilled mammalian protein extraction reagent (M‐PER; #78501, Thermo Fisher Scientific) with cOmplete Mini protease inhibitor cocktail (#11836170001, Roche, Indianapolis, IN, USA) was added to lyse cells. Samples were centrifuged at 4 °C (14 000 × ***g***) for 15 min, and the supernatant was collected. Protein concentrations were adjusted by Coomassie Plus (Bradford) Protein Assay Kit (# 23236, Thermo Fisher Scientific). Samples were heated in NuPAGE Sample Buffer (#NP0008, Thermo Fisher Scientific) containing 2.5% 2‐mercaptoethanol (#M6250, Millipore Sigma) at 70 °C for 10 min and electrophoresed using NuPAGE Bis‐Tris Gels 4–12% (#NP0335BOX, Thermo Fisher Scientific) at 120 V for 1 h. After protein transfer to polyvinylidene difluoride membranes (PVDF; #IPVH00010, Millipore Sigma) using the eBlot^tm^ L1 Fast Wet Transfer System (GenScript, Piscataway, NJ, USA), membranes were blocked with 5% nonfat dry milk in TBST for 1 h and incubated with primary antibodies (NLRP3, 1 : 1000, #15101, Cell Signaling Technology, Danvers, MA, USA; IL‐1β 1 : 1000 #12242, Cell Signaling Technology) at 4 °C overnight followed by labeling with HRP‐conjugated secondary antibodies (Anti‐Rabbit, 1 : 2000, #7074, Cell Signaling Technology; Anti‐Mouse, 1 : 2000, #7076, Cell Signaling Technology) at room temperature for 1 h. Incubated membranes were developed with Trident Pico HRP Western Substrate (#GTX17435, Genetex, Irvine, CA, USA) and recorded with the ECL imaging system ChemiDoc MP (Bio‐Rad Laboratories, Hercules, CA, USA). Coomassie staining of PVDF membranes was used to visualize protein transfer.

### Quantitative polymerase chain reaction (qPCR)

Cells were seeded in 6‐well plate (#657160, Greiner Bio‐One) at 2.5 × 10^5^ cells per well and cultured for 48 h until confluency with 2 mL of media per well. RNA extraction was performed with RNeasy plus mini kit (#75034, Qiagen, Germantown, MD, USA), according to the manufacturer's protocol. cDNA was synthetized with iScript Reverse Transcription Supermix for RT‐qPCR (#1708840, Bio‐Rad). Real‐time PCR was performed with Taqman Gene expression Assay (FAM; #4331182, Thermo Fisher Scientific) using primers *NLRP3* (Hs00918082) *ACTB* (Hs01060665), and TaqMan Fast Advanced Master Mix (#4444556, Thermo Fisher). Assay was performed according to the manufacturer's protocol on quantistudio5 (Applied Biosystems, Waltham, MA, USA) thermal cycler for 40 cycles. The relative levels of mRNA expression were calculated by ΔΔ*C*_T_ method normalized to *ACTB* as endogenous control, with three replicates per group.

### Bioinformatic analysis

Uveal melanoma (TCGA‐UVM) and cutaneous melanoma (TCGA‐SKCM) patients' tumor transcriptome profiling gene expression RNA sequencing data were downloaded from The Cancer Genome Atlas (TCGA) website (https://portal.gdc.cancer.gov/). NLRP3‐ and IL1B‐normalized gene expression values in transcripts per million were extracted from each case file to create a new data frame using rstudio [26]. Group medians were compared using the Mann–Whitney *U* test. Single‐cell transcriptomic data and graphs of RPE/choroid cells were acquired from spectacle website [27] (https://singlecell‐eye.org/app/spectacle/) based on Voigt *et al*. [28] dataset.

### Statistical analysis

Statistical analyses were performed by prism software 5.0 (GraphPad, San Diego, CA, USA). Results were described as mean ± SEM. ANOVA followed by *post hoc* Tukey HSD test was used to compare multiple groups. Statistical significance was achieved when *P* < 0.05.

## Results

### Uveal melanoma has lower NLRP3 expression compared with cutaneous melanoma

**Fig. 1:**
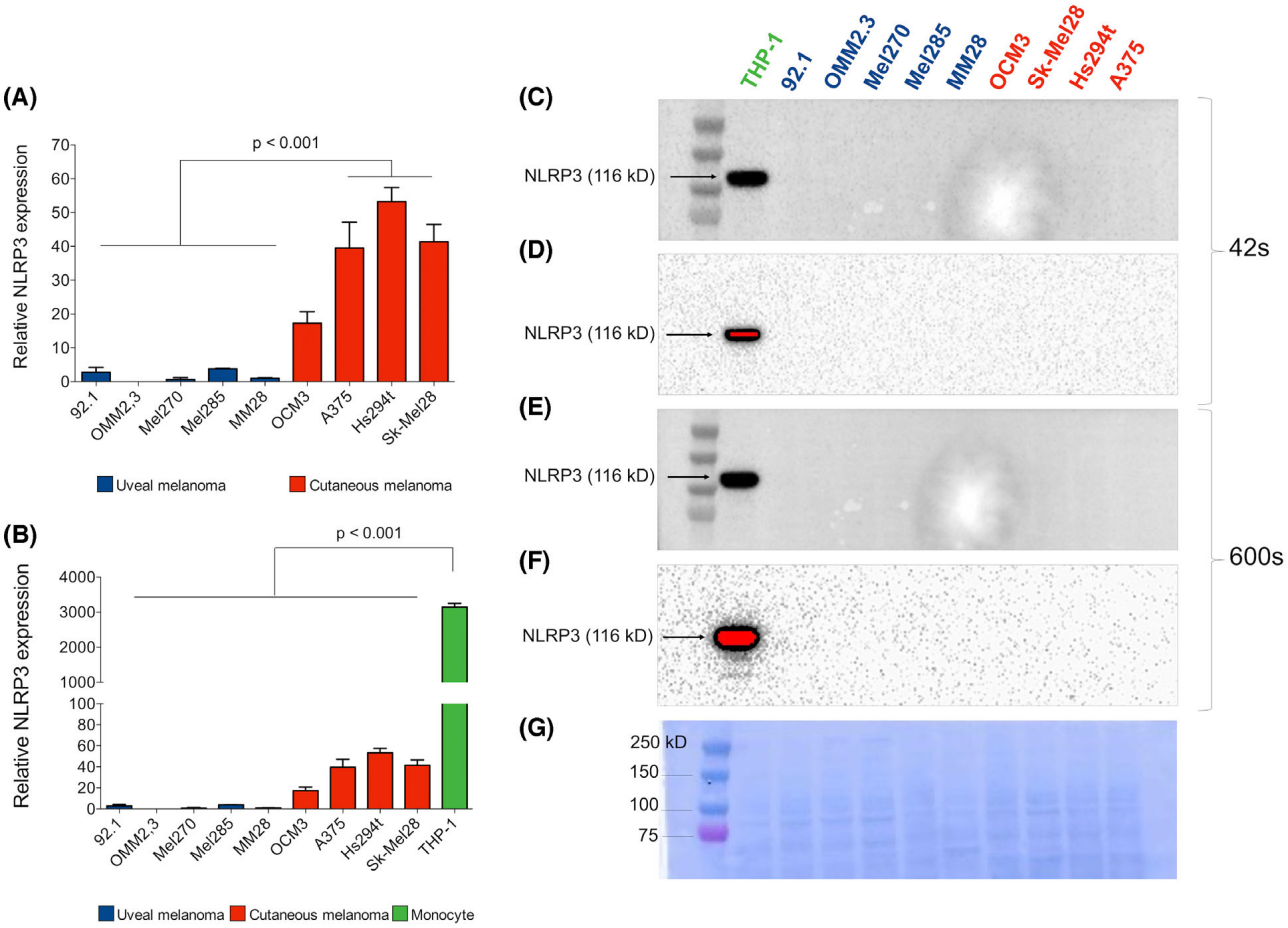
Uveal melanoma has lower NLRP3 expression compared with cutaneous melanoma. (A) NLRP3 mRNA expression levels of UM and CM cell lines measured by qPCR. (B) Comparison of NLRP3 mRNA expression of melanoma cell lines with monocyte cell line THP‐1. (C, D) NLRP3 protein expression analysis by western blotting in melanoma cell lines and the positive control THP‐1 at 42 s of membrane exposure. Saturated pixels are highlighted in red. (E, F) Overexposure of the same membrane at 10 min. (G) Coomassie blue staining of the membrane. Statistical significance was analyzed with ANOVA followed by *post hoc* Tukey HSD test. All values are expressed as mean ± SEM (*n* = 3).

qPCR analysis showed that NLRP3 gene expression by the five UM cell lines was significantly lower in comparison with CM cell lines (*P* < 0.001; Fig. 1A). Further, both uveal and cutaneous melanoma showed very low NLRP3 mRNA levels when compared to the monocyte cell line THP‐1 (*P* < 0.001; Fig. 1B). Overall protein levels of NLRP3 by WB using a previously validated specific and very sensitive antibody [29], failed to detect any signal even after prolonged exposure (10 min) on any melanoma cell line, suggesting that the NLRP3 protein levels in melanoma, if present, are extremely low (Fig. 1C–G).

### Uveal melanoma has minimal baseline secretion of IL‐1β

**Fig. 2:**
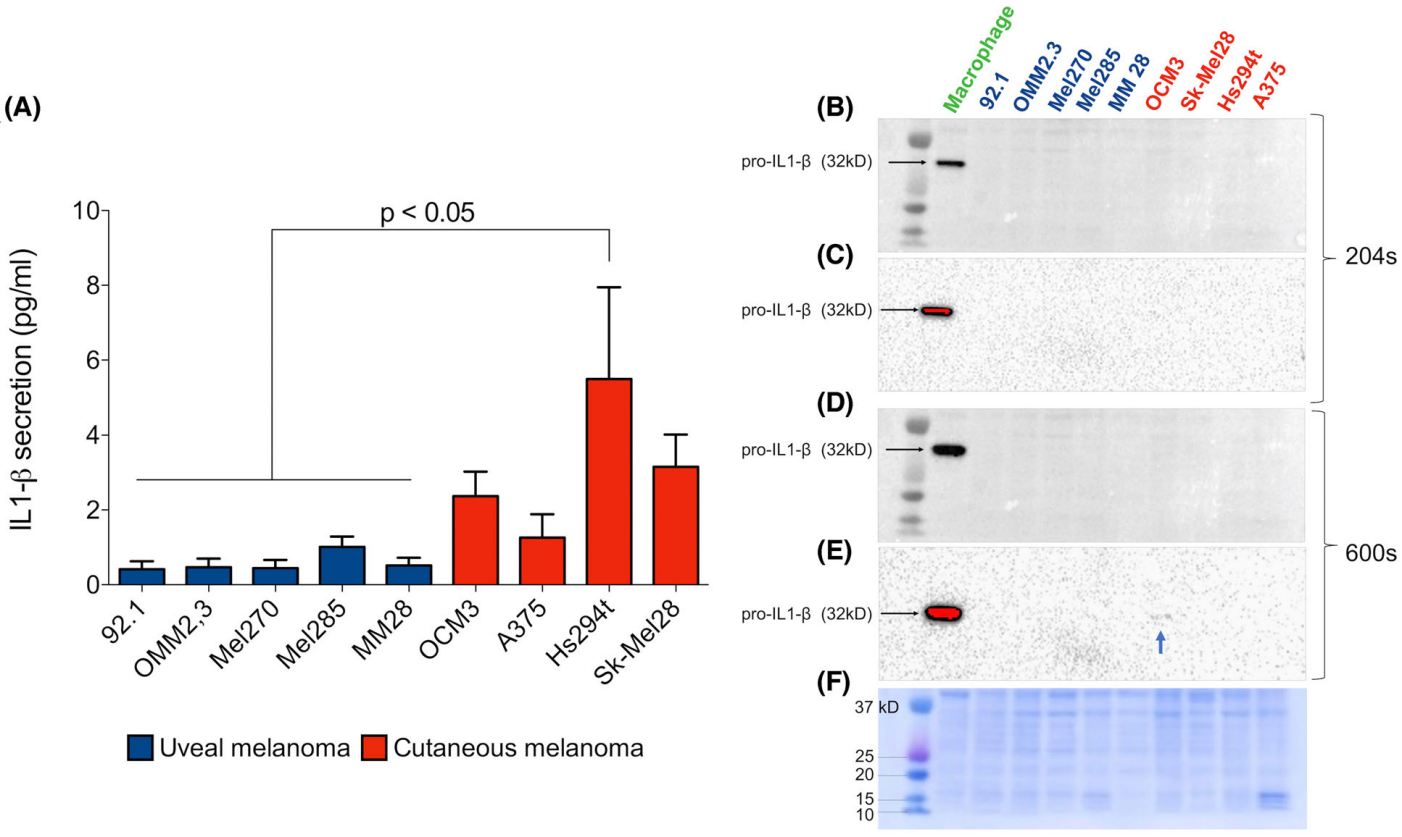
Uveal melanoma has minimal baseline secretion of IL‐1β. (A) Baseline secretion levels of IL‐1β by melanoma cell lines. (B, C) IL‐1β precursor (pro‐IL‐1β) protein expression analysis by WB in melanoma cell lines and the positive control (THP‐1‐derived macrophage) at 204 s of membrane exposure. Saturated pixels are highlighted in red. (D, E) Overexposure of the same membrane at 10 min. A faint band on OCM3 is indicated by the blue arrow. (F) Coomassie blue staining of the membrane. Statistical significance was analyzed with ANOVA followed by *post hoc* Tukey HSD test. All values are expressed as mean ± SEM (*n* = 5).

We proceeded to investigate the expression levels of IL‐1β by WB and ELISA using THP‐1‐derived macrophage as positive control (Fig. 2). WB with equal protein loading (Fig. 2F) showed no IL‐1β signal on any cell line at 204 s (Fig. 2B,C), and after prolonged exposure, only a faint band on OCM3 cell line was revealed (Fig. 2D,E). ELISA detected low levels of IL‐1β in all cell lines (Fig. 2A), with UM cell lines secreting lower levels compared with CM cells, with significant difference when compared to the highest expressing CM cell line (Hs294t, *P* < 0.05). Since the recent report by Tengesdal *et al*. suggested that the CM cell lines may have significantly higher IL‐1β secretion than what we detected, we further reviewed the literature to address baseline IL‐1β secretion by melanoma cell lines (Table 1). The levels reported were overall low, similar to our findings, with a few exceptions, especially with the cell line 1205Lu.

**Table 1:** IL‐1ß secretion levels in melanoma cell lines. Summary of the reported values of IL‐1ß secretion of melanoma cell lines measured by ELISA. Some of the values shown are approximated based on the original data.

Cell line	IL1B (pg·mL^−1^)	Type	References	Cell line	IL1B (pg·mL^−1^)	Type	References
1205Lu	> 50	Cutaneous	Okamoto, 2010 [30]	MA2058	0	Cutaneous	Gehrke, 2014 [31]
1205Lu	120	Cutaneous	Liu, 2013 [32]	Memel 82	0	Cutaneous	Gehrke, 2014 [31]
1205Lu	120	Cutaneous	Ellis, 2011 [33]	MeWo	0.15–0.2	Cutaneous	Rovera, 2022 [34]
1205Lu	0.1–0.15	Cutaneous	Rovera, 2022 [34]	MM001	0.15–0.2	Cutaneous	Rovera, 2022 [34]
1205lu	300–400	Cutaneous	Tengesdal, 2021 [23]	MM029	0	Cutaneous	Rovera, 2022 [34]
1205Lu	600–800	Cutaneous	Tengesdal, 2021 [24]	MM074	0.15–0.2	Cutaneous	Rovera, 2022 [34]
501Mel	0	Cutaneous	Young, 2017 [35]	MM099	0.15–0.2	Cutaneous	Rovera, 2022 [34]
501Mel	0.15–0.2	Cutaneous	Rovera, 2022 [34]	MM485	0	Cutaneous	Young, 2017 [35]
888mel	0	Cutaneous	Young, 2017 [35]	MNC1	0.05–0.1	Cutaneous	Rovera, 2022 [34]
A357^a^	10–15	Cutaneous	Tengesdal, 2021 [24]	MUM2B	75–100	Uveal	Triozzi, 2011 [36]
A375	9	Cutaneous	Okamoto, 2010 [30]	MV3	0	Cutaneous	Young, 2017 [35]
A375	0	Cutaneous	Gehrke, 2014 [31]	OCM1	0–25	Uveal	Triozzi, 2011 [36]
A375	0	Cutaneous	Young, 2017 [35]	SBCI2	< 0.05	Cutaneous	Rovera, 2022 [34]
DO4	0	Cutaneous	Young, 2017 [35]	SK‐MEL28	0.15–0.2	Cutaneous	Rovera, 2022 [34]
EB16Mel	2050	Cutaneous	Kholmanskikh, 2010 [37]	WM115	< 2	Cutaneous	Okamoto, 2010 [30]
HS294T	20	Cutaneous	Okamoto, 2010 [30]	WM115	0	Cutaneous	Liu, 2013 [32]
Hs294t	80	Cutaneous	Liu, 2013 [32]	WM1361	0	Cutaneous	Young, 2017 [35]
Hs294T	25–30	Cutaneous	Ellis, 2011 [33]	WM1552C	< 2	Cutaneous	Okamoto, 2010 [30]
Kul84‐Mel	< 3	Cutaneous	Kholmanskikh, 2010 [37]	WM164	0.15–0.2	Cutaneous	Rovera, 2022 [34]
LB2259‐Mel	< 3	Cutaneous	Kholmanskikh, 2010 [37]	WM164	0	Cutaneous	Young, 2017 [35]
LB2439‐Mel	< 3	Cutaneous	Kholmanskikh, 2010 [37]	WM2032	< 0.05	Cutaneous	Rovera, 2022 [34]
M000 291	0	Cutaneous	Gehrke, 2014 [31]	WM266‐4	25–30	Cutaneous	Serini, 2020 [38]
M010 119	0	Cutaneous	Gehrke, 2014 [31]	WM266‐4	0	Cutaneous	Young, 2017 [35]
M010 817	0	Cutaneous	Gehrke, 2014 [31]	WM278	< 2	Cutaneous	Okamoto, 2010 [30]
M060 125	0	Cutaneous	Gehrke, 2014 [31]	WM35	< 2	Cutaneous	Okamoto, 2010 [30]
M080 228	0	Cutaneous	Gehrke, 2014 [31]	WM35	0	Cutaneous	Liu, 2013 [32]
M080 306	0	Cutaneous	Gehrke, 2014 [31]	WM75	< 2	Cutaneous	Okamoto, 2010 [30]
M080 904	0	Cutaneous	Gehrke, 2014 [31]	WM793	< 0.05	Cutaneous	Rovera, 2022 [34]
M081 021	0	Cutaneous	Gehrke, 2014 [31]	WM793B	44	Cutaneous	Okamoto, 2010 [30]
M081028	0	Cutaneous	Gehrke, 2014 [31]	WM852	0	Cutaneous	Young, 2017 [35]

^a^ Likely a typing mistake on the original paper, probably referring to the cell line A375.

### Human samples gene expression analysis reveals the low expression of NLRP3 and IL‐1β on uveal melanoma and normal choroidal melanocytes

**Fig. 3:**
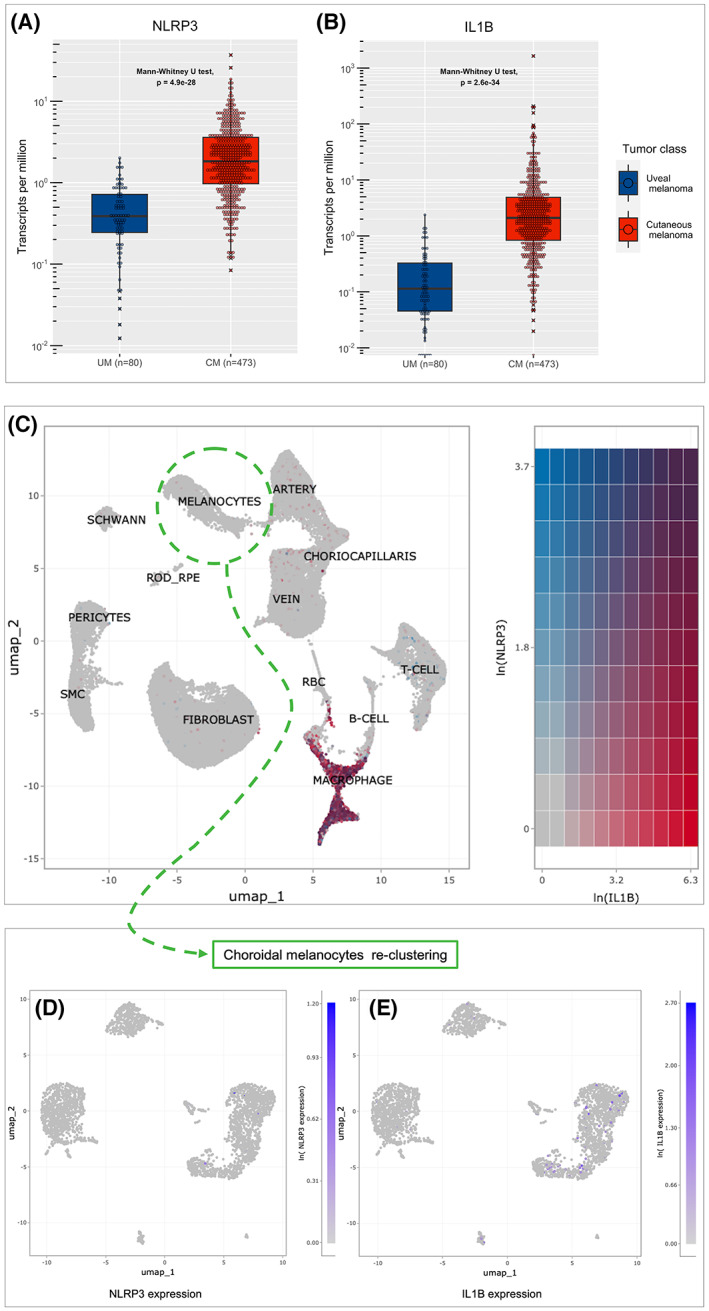
Bioinformatic analysis from human samples reveals the low expression of NLRP3 and IL‐1β on uveal melanoma samples and normal choroidal melanocytes. (A, B) Analysis of UM and CM gene expression levels from the Cancer Genome Atlas (TCGA) database. UM cases show significantly lower levels of NLRP3 and IL‐1β compared with CM. (C) Single‐cell clustering of normal human choroidal tissue showing the heatmap of NLRP3 (blue) and IL‐1β (red) co‐expression. Melanocytes (green circle) have low expression of both genes. (D, E) Reclustering of melanocytes reveals that most cells do not express NLRP3 or IL‐1β. Statistical significance was analyzed with Mann–Whitney *U* test. Outliers are marked with X.

To verify whether our *in vitro* cell line results are also observed in whole tumor samples, we compared human melanoma samples gene expression data available from The Cancer Genome Atlas (TCGA) database. UM had significantly lower gene expression levels of NLRP3 and IL‐1β compared with CM (Fig. 3A,B). Additionally, we explored if the NLRP3‐IL‐1β axis is activated on normal choroidal melanocytes, since these are the cells that originate UM. Transcriptomic analysis of RPE/choroid cells acquired from the Spectacle single‐cell atlas [27] reveals that choroidal melanocytes do not express NLRP3 or IL‐1β (Fig. 3C). Reclustering of choroidal melanocytes further demonstrates that the vast majority of cells do not express NLRP3 or IL1β (Fig. 3D,E).

## Discussion

During the last decade, the role of inflammasomes in tumorigenesis, antitumor immunity, and response to cancer therapy has been investigated in many cancer models, with conflicting findings [25, 39, 40, 41]. While inflammasome‐associated IL‐1β secretion by tumor‐associated macrophages and cancer‐associated fibroblasts may promote tumor growth and metastasis by suppressing antitumor immunity [42, 43, 44], inflammasome activation also enhances antitumor activity of CD8^+^ T cells and NK cells through IL‐18, promotes tumor infiltration of lymphocytes, and strengthens the response to checkpoint inhibitors [45, 46, 47, 48].

NLRP3 inflammasome was investigated specifically in CM by other authors as well, with contrasting results. Lee *et al*. [49] demonstrated that NLRP3 inflammasome knockout or pharmacological inhibition reduced the ability of macrophages to promote migration and invasion of CM cells. Also, an increased expression of NLRP3 inflammasome‐related genes in CM tumor samples was associated with low or no response to immune checkpoint therapy [50]. Conversely, a high expression of NLRP3 inflammasome‐related genes was associated with increased CM overall survival [50, 51] and higher tumor immune infiltration of B cells, CD8^+^ T cells, and neutrophils [51]. In addition, NLRP3 inflammasome activation by nigericin induced pyroptotic cell death on BRAF V600 inhibitor‐resistant CM tumor cells [52].

To date, there have been no reports of the NLRP3 inflammasome‐IL‐1β axis in UM. In this setting, we evaluated the baseline levels of IL‐1β secreted by five different UM cell lines and four different CM cell lines. Nonstimulated secretion was overall low, on the picogram scale (Fig. 2A). In addition, UM cells showed significantly lower levels of IL‐1β secretion compared with the CM cell line Hs294t (*P* < 0.05), which might indicate an even lower potential of UM to recruit immune cells to the tumor site [23, 24, 53]. The levels reported in our study for the CM cell lines we used were comparable to other published literature (Table 1) and in accordance with Gehrke *et al*. [31] that showed no IL‐1β secretion by CM cell lines. Most skin melanoma cell lines express low levels of IL‐1β, with only few exceptions, particularly the cell line 1205Lu. It is worth mentioning that this specific cell line is potentially contaminated and might not reflect the original tumor characteristics [54, 55]. These data are in accordance with a recently published paper that demonstrated through single‐cell RNA‐seq that myeloid cells, and not tumor cells, are the primary source of IL‐1β in solid cancers [56].

Since we have investigated both primary and metastatic cell lines (Table S1), these data suggest that there is no meaningful endogenous IL‐1β secretion by melanoma cell lines regardless of origin. Collectively, our findings indicate that melanoma‐derived IL‐1β might represent only a small fraction of the IL‐1β within the tumor microenvironment and targeting NLRP3 would affect primarily immune rather than tumor cells. In addition, we demonstrate that UM cell lines have even lower NLRP3 gene expression and IL‐1β secretion than CM cell lines, which might help explain, at least partially, the differences in UM response to immune checkpoint inhibitors [9, 14, 15, 16, 17, 18, 19, 20, 21].

To further explore the topic, we conducted a bioinformatic analysis of human tumor samples from the TCGA database. The TCGA is a public available database of cancer multiomics data with over 20 000 cases of primary cancer and matched normal samples. UM samples had significatively lower expression levels of both NLRP3 and IL‐1β compared with CM samples. Additionally, we investigated the gene expression of normal choroidal melanocytes through the Spectacle single‐cell atlas [27] based on a dataset with 37 070 cells including 2939 melanocytes [28] and showed that these cells that originate UM also do not express NLRP3 or IL1B.

A limitation of our study is the sole use of *in vitro* and bioinformatic approaches. Although the use of animal models could provide data on the interaction between tumor and immune cells, the primary objective of this work was to determine the endogenous baseline NLRP3 inflammasome levels and IL‐1β secretion of CM and UM. Another limitation of our study is that we only investigated established cell lines, which could differ from the original tumor cells. On the contrary, the use of nine different cell lines with concordant observations and the validation of our findings with human tumor samples data give us confidence in our results.

To conclude, our work shows evidence of very low NLRP3 expression and IL‐1β secretion by melanoma cells and highlights the differences between cutaneous and uveal melanoma, providing data that may contribute to future melanoma translational research.

## Conflict of interest

The authors declare no conflict of interest.

## Author contributions

VSMCC, NEE, and DGV conceived the paper. VSMCC and NEE designed the experiments. VSMCC, NEE, ZY, DPN, and TN conducted the experiments. VSMCC and NEE performed data analysis. VSMCC drafted the paper. EG and IKK contributed to the review and editing. DGV supervised the project. All authors contributed to the edits and approval of the final paper.

## Supporting information


**Table S1.** Melanoma cell line characteristics.

## Data Availability

The datasets used and/or analyzed during the current study are available from the corresponding author on reasonable request.
