# Supplementary material for: The NLRP3 inflammasome – interleukin 1β axis in uveal melanoma
Source: FEBS Open Bio. 2023 Feb 12;13(3):545–55. doi: 10.1002/2211-5463.13566 (PMC9989921; doi:10.1002/2211-5463.13566)
Supplement: Supplementary file 1 — Table S1. Melanoma cell line characteristics. [file FEB4-13-545-s001.docx]

**Supplemental Table S1 - Melanoma cell lines characteristics**

| **Cell Line** | **92.1** | **Mel 270** | **OMM2.3** | **Mel 285** | **MM28** | **OCM3** | **A375** | **Hs294T** | **Sk-Mel 28** |
| --- | --- | --- | --- | --- | --- | --- | --- | --- | --- |
| **Origin** | UM | UM | UM | UM | UM | CM[57] | CM | CM | CM |
| **Tissue** | Primary | Primary | Liver  Metastasis | Primary | Liver  Metastasis | Primary | Primary | Lymph node Metastasis | Lymph node Metastasis |
| **GNAQ** | p.Q209L[57] | p.Q209L[57] | p.Q209L[57] | WT[58] | WT[59] | WT[57] | - | - | - |
| **GNA11** | WT[57] | WT[57] | WT[57] | WT[58] | c.626A>T[59] | WT[57] | - | - | - |
| **BRAF** | WT[57] | WT[57] | WT[57] | - | - | p.V600E[60] | p.V600E[61] | p.V600E[61] | p.V600E[61] |
| **NRAS** | WT[57] | WT[57] | WT[57] | - | - | WT[57] | WT[61] | WT[61] | WT[61] |
| **BAP1 Mutation** | WT[58] | WT[58] | - | WT[59] | c.1881C>A[59] | - | - | - | - |
| **BAP1 IHC** | Positive[58] | Positive[58] | Positive[58] | Negative[58] | Negative[59] | Positive[62] | - | - | - |
| **EIF1AX** | c.17G>A [58] | WT [58] | WT[58] | WT[58] | WT [59] | - | - | - | - |
| **Chromosome 3** | Disomy[58] | Disomy[58] | Disomy[58] | Disomy[58] | Monosomy[59] | Disomy[63] | - | - | - |
| **Chromosome 8** | Gain 8q[58] | Disomy 8q[58] | Disomy 8q[58] | Disomy 8q[58] | Gain 8q[59] | - | - | - | - |
| **Reference**[64] | De Waard-  Siebinga[58] | Verbik[65] | Verbik[65] | Verbik[65] | Amirouchene-  Angelozzi[59] | Huang[66] | Giard[67] | Fogh[68] | Carey[69] |
